# Characterization of organophosphatic brachiopod shells: spectroscopic assessment of collagen matrix and biomineral components†

**DOI:** 10.1039/d0ra07523j

**Published:** 2020-10-20

**Authors:** Oluwatoosin B. A. Agbaje, Simon C. George, Zhifei Zhang, Glenn A. Brock, Lars E. Holmer

**Affiliations:** Department of Earth Sciences, Palaeobiology, Uppsala University Uppsala Sweden toosin.agbaje@mq.edu.au toosin91014@gmail.com; Department of Earth and Environmental Sciences and MQ Marine Research Centre, Macquarie University Sydney Australia; Department of Biological Sciences, Macquarie University Sydney Australia; State Key Laboratory of Continental Dynamics, Shaanxi Key Laboratory of Early Life & Environments, Department of Geology, Northwest University Xi'an 710069 China

## Abstract

The shells of linguloid brachiopods such as *Lingula* and *Discinisca* are inorganic–organic nanocomposites with a mineral phase of calcium phosphate (Ca-phosphate). Collagen, the main extracellular matrix in Ca-phosphatic vertebrate skeletons, has not previously been clearly resolved at the molecular level in organophosphatic brachiopods. Here, modern and recently-alive linguliform brachiopod shells of *Lingula* and *Discinisca* have been studied by microRaman spectroscopy, Fourier transform infrared spectroscopy, field emission gun scanning electron microscopy, and thermal gravimetric analysis. For the first time, biomineralized collagen matrix and Ca-phosphate components were simultaneously identified, showing that the collagen matrix is an important moiety in organophosphatic brachiopod shells, in addition to prevalent chitin. Stabilized nanosized apatitic biominerals (up to ∼50 nm) permeate the framework of organic fibrils. There is a ∼2.5-fold higher wt% of carbonate (CO_3_^2−^) in *Lingula versus Discinisca* shells. Both microRaman spectroscopy and infrared spectra show transient amorphous Ca-phosphate and octacalcium phosphate components. For the first time, trivalent moieties at ∼1660 cm^−1^ and divalent moieties at ∼1690 cm^−1^ in the amide I spectral region were identified. These are related to collagen cross-links that are abundant in mineralized tissues, and could be important features in the biostructural and mechanical properties of Ca-phosphate shell biominerals. This work provides a critical new understanding of organophosphatic brachiopod shells, which are some of the earliest examples of biomineralization in still-living animals that appeared in the Cambrian radiation.

## Abstract 2

The shells of linguloid brachiopods such as *Lingula* and *Discinisca* are inorganic–organic nanocomposites with a mineral phase of calcium phosphate (Ca-phosphate).

## Graphical abstract

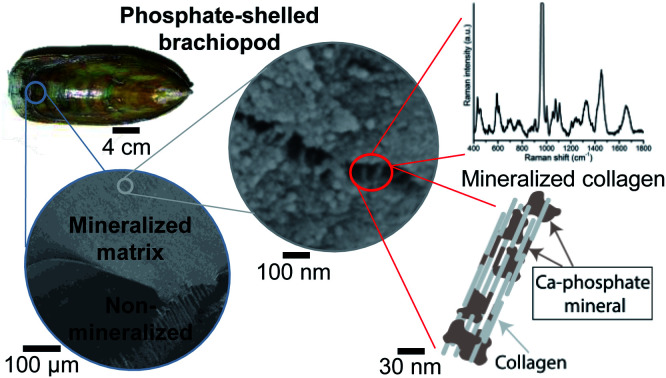

## 1. Introduction

Brachiopods are a phylum of sessile, filter-feeding, epibenthic lophophorates that enclose their soft parts between two robust biomineralized shells. The group originated during the Cambrian radiation, dominated Palaeozoic marine benthic ecosystems, and some lineages range through the entire 541 million years of the Phanerozoic Eon.^1^ The shells have a distinctive combination of a nanoscale inorganic matrix embedded within organic macromolecules.^2–4^ The phylum consist of three distinctive subphyla characterized by organocalcitic (Craniiformea and Rhynchonelliformea) and organophosphatic (Linguliformea) shells, which are of interest to materials science due to the hierarchically organized composites that are lightweight and have unique combinations of strength and toughness.^4^ The hierarchical architecture of the two types of shell composition primarily behave as a matrix and as a reinforcement: the former consists of organic macromolecules, while the latter is an inorganic biomineral. The hybrid components in biological structural materials enhance the strength of shell architecture as well as functional tasks. The organic matrix in organocalcitic shells represent a very small fraction, about 2 wt%, whereas in organophosphatic shells, the fibres are organic biopolymers reinforced with Ca-phosphate nanoparticles to form a fibrous biocomposite.^3,4^

The goal of this work is to investigate the organophosphatic shell of modern and recently-alive linguloid brachiopods, so as to improve understanding of biopolymers and to assess the degree of integrated biogenic mineral components. Several studies have examined the phosphatic shells of *Lingula* and *Discinisca*: shell biominerals normally contain membranes of protein and mineralized chitin, and consists of spherular apatite occluded in glycosaminoglycans with varying amounts of mineralization.^5–7^ The glycosaminoglycans have been proposed to influence the biomineralization of brachiopod shells,^8^ in the same way as for vertebrate bones^9^ with mineral hydroxyapatite (Ca_10_(PO_4_)_6_(OH)_2_). There is basic agreement that vertebrate bones and linguliform shells are apatitic.^4,10^ However, other workers have observed that living linguloid shells are composed of carbonate-substituted fluorapatite,^11^ similar to the geological mineral francolite. Also, differences between organic participation in phosphate-shelled brachiopods and vertebrate bone matrices have been identified by solid state-nuclear magnetic resonance.^8^ Organic chitin dominates, especially, in Ca-phosphate shell biominerals.^4^ However, in vertebrate bone, carbonate apatite is embedded in an organic collagen framework (with other minor constituents) to reinforce their mechanical strength and flexibility.^12,13^ In collagen, the amide group of glycine is highly protected, in that higher amounts of glycine plays a crucial role in the conformation of an uncommon secondary structure called 3_10_-helix or triple helix, whereas amino acid analyses of phosphatic shells reveals low glycine and high alanine residues.^3,6,14^

Typical analyses of the chemical composition of bivalve mollusc shells involves various demineralization methods, including several steps such as grinding shell biominerals into a powder.^6,15^ This step was excluded in the present study to probe chemical environments within the intact brachiopod shells. Less attention has been devoted to the (typically) extracellular matrix, mainly collagen, and the possible interactions of the matrix with the major components of apatite shells.^3,14,16^ The organic content occluded in the mineralized matrix and comprehensive identification of the individual components of the organic matrix remains a considerable analytical challenge. The main aim of this investigation is to explore the interaction of shell protein and inorganic components in the shells of recent organophosphatic brachiopods by synchronous analysis.

Vibrational spectroscopy, including microRaman spectroscopy and attenuated total reflectance Fourier transform infrared (ATR-FTIR) spectroscopy, are powerful non-destructive techniques suitable for investigation of the molecular structure of biominerals and biomaterials.^17–19^ These techniques have been used in this study to characterize the shell composition of linguliform brachiopods, and to compare the data with the known structure of type I collagen, sulphated glycosaminoglycan (chondroitin sulfate A) and polysaccharides, that is, chitin and chitosan. One advantage of this approach is that it enables the simultaneous measuring of covalently-bonded atoms of organic macromolecules and the inorganic matrix, so as to provide a complete picture of the biominerals. Both FTIR and microRaman spectroscopy have been extensively used to assess biomineralized tissue heterogeneity, and offer similar and, in part, complementary information.^19–22^ Raman spectroscopy suffers from an inferior signal to noise ratio when compared to FTIR spectroscopy, but is based on light scattering rather than absorption.^19^ For instance, water has a very weak Raman scattering cross section.^23^ Biomolecules can be studied in an aqueous analytical environment, thus enabling *in situ* recording of high quality Raman spectra of biomaterials. Another specific advantage of Raman spectroscopy is that it uses a microscope to focus the laser beam, enabling analysis of biologically-important localities such as individual lamellae, individual cement lines, suture regions.^24,25^ Previous studies have used FTIR and Raman spectroscopy to spatially resolve, for instance, the components of bone.^20,26–28^ In Raman spectroscopy the most prominent phosphate region, *v*_1_PO_4_^3−^, is somewhere between 945 and 965 cm^−1^, but the exact position is sensitive to various Ca-phosphate frequencies such as amorphous calcium phosphate (ACP), octacalcium phosphate (OCP), carbonated hydroxyapatite (CAP), hydroxyapatite (HAP) and tricalcium phosphate (TCP).^24,29^ The FTIR and Raman signals associated with collagen and non-collagenous organic components at 1200–1343 cm^−1^ (amide III), 1580–1720 cm^−1^ (amide I), and 2800–3050 cm^−1^ (C–H stretch) are of particular interest for the recognition of apatite matrices.^20,25,27^

This study aims to address long standing issues related to the framework dynamics of organic constituents and mineral components in invertebrate (specifically brachiopod) shell biominerals, issues that are similar to those for vertebrate bones and teeth. MicroRaman spectroscopy is complemented by ATR-FTIR spectroscopy, field gun emission scanning electron microscope (FEG-SEM) imaging, and thermal gravimetric analysis (TGA) so as to characterise the chemical composition of brachiopod shells. While recent work has provided evidence of core fibres, composed of a chitin matrix (Agbaje *et al.* unpublished data), the resulting data in this work provide information on the proteinaceous component in the phosphate-shelled brachiopods, and allows comparison with propensities for the type I collagen protein motif, sulphated glycosaminoglycan and polysaccharides.

## 2. Samples and experimental techniques

### 2.1 Materials

Specimens of recently-alive and modern/living organophosphatic brachiopods, *Lingula anatina* (Lamarck, 1801) and *Discinisca tenuis* (Sowerby, 1847) were sampled and investigated. Modern/living *L. anatina* was collected from the Bay of Guangxi, China and preserved in 10% formalin prior to analyses. Although invertebrates typically require 4% formalin to preserve their hybrid composite materials from distortion or deterioration, 10% is adequate since the size/volume of the sample was considered in estimation of final concentration.^30^ Neary *et al.*^8^ fixed *Lingula anatina* and other biominerals in ethanol for weeks, and the solvent had no effect on these samples. For this work, the effect of 10% formalin cannot be greatly different from those described by the latter authors. Shells of recently alive *L. anatina* and *D. tenuis* were collected from Moreton Bay, Queensland, Australia and Walvis Bay Namibia, respectively.

### 2.2 Sample preparation

Organophosphatic shells of modern/living (ML) and recently alive (RL) specimens of *L. anatina*, and recently alive shells of *D. tenuis* (ESI Fig. S1†), were cleaned with a scalpel and then washed with Milli-Q water to remove external contaminants. Samples were randomly broken into a few mm-sized pieces, and soaked in hydrogen peroxide (35%; Chem-Supply, UN 2014) for about 2.5 hours to remove extraneous surface-absorbed organic matter. Subsequent preparation involved bleaching in 5% sodium hydroxide and 35% hydrogen peroxide (1 : 2) for a few minutes (≤45 minutes) to remove pigments with intense fluorescence, which make the acquisition of Raman spectra impossible. It has previously been shown that exposure of shell biominerals to solutions of these chemicals for <3 hours causes no alteration to their composition or structure.^15,31,32^ Even after 240 hours (ten days) of oxidation with a less persistent oxidant, hydrogen peroxide, ∼50% of the original organic concentration persisted within the shell biomineral powders.^31^ In the presence of biominerals with carbonate, hydrogen peroxide became less effective at oxidizing organic compounds; also hydrogen peroxide is thermodynamically unstable, decomposing into water and oxygen.^31^ Samples were washed in Milli-Q water until a pH of ∼6.8 was obtained, and were then rinsed briefly with cold acetone twice, then air dried at room temperature. Commercially available type I collagen, extracted from rat tail (Sigma-Aldrich; C7661), chondroitin sulfate A sodium salt from bovine trachea (Sigma-Aldrich; C9819), chitin extracted from shrimps (Sigma-Aldrich; C7170) and chitosan extracted from shrimp shells (Sigma-Aldrich; C3646) were used as standards.

### 2.3 Analytical methods

TGA data, FEG-SEM data, microRaman spectra and ATR-FTIR spectra were acquired for all samples. For microRaman spectroscopy, a Horiba Jobin Yvon LabRAM HR Evolution spectrometer equipped with a charge-coupled device (CCD) detector, an Olympus BX41 microscope and an automated *x*–*y* stage, was used to examine the chemical composition of the shells. An excitation wavelength of 633 nm was used, and a power of ∼10 mW was focused on the sample through a 50× long-working distance microscope objective. Raman scattered light was dispersed by a grating with 600 grooves per mm, and a slit width of 100 μm was used. The spectra were recorded in the range 400–1800 cm^−1^ with an integration time of 40 s (average time), 10 accumulations, and a delay time of 3 s, so as to reduce fluorescence and improve the signal to noise ratio. A confocal arrangement with a 300 μm pinhole was used. By reference to the work of Tabaksblat *et al.*,^33^ a penetration depth of the laser in the order of 6 μm into the sample studied is expected. In this work, the fluorescence signal was more pronounced for a grating with 1800 grooves per mm, and in some cases the unbleached shell samples with a prominent periostracum (an unmineralized layer) had partially masked Raman signals (not shown). The difference between spectra recorded at different times after Ne–He laser illumination provided a good estimate of the extent of the fluorescence signal, and how to avoid it. Sixteen spectra were recorded for all samples. The Raman spectrometer was calibrated before and after measurement using the 520.69–520.72 cm^−1^ peak of a silicon wafer.

An iS10 Thermo Nicolet Smart Performer ATR-FTIR spectrometer (Nicolet, MA, USA) was used at a resolution of 2 cm^−1^ and 64 accumulations. An angle of incidence of 45° and an optical velocity of ∼0.4747 were used. A depth of penetration of 2–3 μm was used to record the data. The range of frequencies was 4000–600 cm^−1^ and background spectra were measured at the start of each analysis.

For TGA, about 4 mg of sample was heated at a rate of 10 °C min^−1^ from 25 °C to 900 °C using a TGA 2050 Thermogravimetric analyzer (TA Instruments, USA) equipped with differential thermal gravimetric (DTG) analyzer. The analyses were recorded twice for each sample.

Each sample was mounted on an aluminium SEM sample holder, and was gold coated for imaging with a JEOL JSM-7100F field emission gun scanning electron microscope (FEG-SEM) at an electron energy of 10 kV and a 10 mm working distance.

### 2.4 Data analysis

All data were analysed using OriginPro 2017 (OriginLab) equipped with an additional peak-fitting module, and are presented as normalized intensities. The different components of each spectrum were determined by overlapping Gaussian curves optimized by the successive iteration in the components through a second derivative. Peaks of each spectrum were considered fitted when they converged with an *R*^2^ value of 0.995 or greater.

Where the relative numbers are important, FTIR metrics were preferred to investigate the maturity of the collagen cross-link ratio, which was calculated by taking the integral ratio of the areas of sub-peaks at ∼1660 cm^−1^ and ∼1690 cm^−1^ under the amide I peak.^20,27^ Individual Raman measurements of apatite biominerals in the amide I region have lower signal-to-noise ratios and are less precise than single infrared measurements.^20,27^ However, Raman spectroscopy offers more intense peaks in the phosphate *v*_1_ (*v*_1_PO_4_^3−^) mode, in the range 990–900 cm^−1^, compared to FTIR spectroscopy, enabling analysis of biologically-important parameters such as the mineral components and mineral crystallinity.^19,24,34^ The *v*_1_PO_4_^3−^ peak envelope in biominerals is asymmetric and consists of closely-spaced, incompletely resolved peaks.^29,34^ Peak fitting of the *v*_1_PO_4_^3−^ phosphate region permits interpretation of the composition and the mineral crystallinity of each spot. The underlying *v*_1_PO_4_^3−^ peaks at about 950 cm^−1^ (ACP), 955 cm^−1^ (OCP), 964 cm^−1^ (HAP) and 974 cm^−1^ (TCP) were used to determine the ACP : OCP, ACP : HAP and ACP : TCP area ratios.^24,35^ The underlying FTIR peaks in the 900–1200 cm^−1^ region were also fitted.

## 3. Results

### 3.1 Shell structure and organic–inorganic composite of linguliform brachiopods

**Fig. 1:**
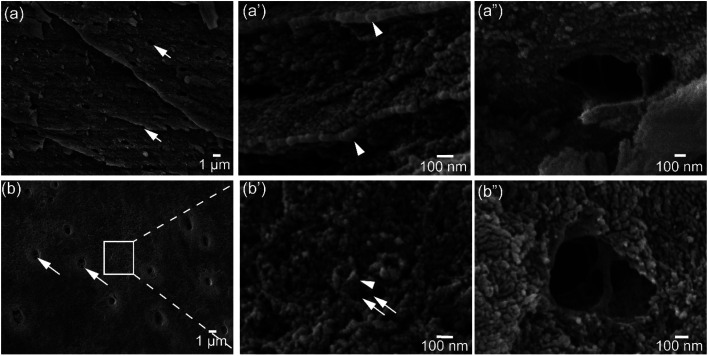
Field emission gun scanning electron microscope images of hydrogen peroxide-treated brachiopod shells. (a) Representative external shell surface of recent *Lingula anatina* showing organic fibrils across the pores (arrows) and (a′) cross-section. Organic fibrils are visible in (a′′). (b) External surface of *Discinisca tenuis* shell with pores, expanded in (b′) to show nanoparticle granules. The arrows in (b) depict collagen-like organic fibrils across the pores and more visible in (b′′). See text for details. The length and the width of the pores for both *L. anatina* and *D. tenuis* are 220–250 nm and 170–190 nm, respectively. The arrowheads in (a′) and (b′) show the calcium phosphate nanoparticles with spherical and elongated shapes, and nanoparticle sizes on the order of 45–65 nm (*Discinisca*) and 45–80 nm (*Lingula*) arranged around organic fibrils.

Architectural features of the shell samples are shown as structural images from FEG-SEM for the shells of *L. anatina* (Fig. 1a) and *D. tenuis* (Fig. 1b). The inorganic component of the external surface of shell biominerals can be considered to be an assembly of distinct levels of hierarchical structural units consisting of arrays of organic matrix fibres. The fibres are composed of stacks of growth units made of Ca-phosphate nanoparticles, with an average diameter of 45–80 nm for *L. anatina* shells and 45–65 nm for *D. tenuis* shell. The ultrastructural architectures reveal pores which are of irregular shape but of a characteristic size and spacing (Fig. 1). Pores are one of the important characteristics of the ultrastructure of shell biominerals.^36^ The nanometric diameters of the pores for the shells of *L. anatina* and *D. tenuis* are in the order of 220–250 nm long and 170–190 nm wide, filled with organic fibrils. Such fibrils are different from crystalline chitin fibrils, but correspond to a collagen-type matrix.^5,6,37^

**Fig. 2:**
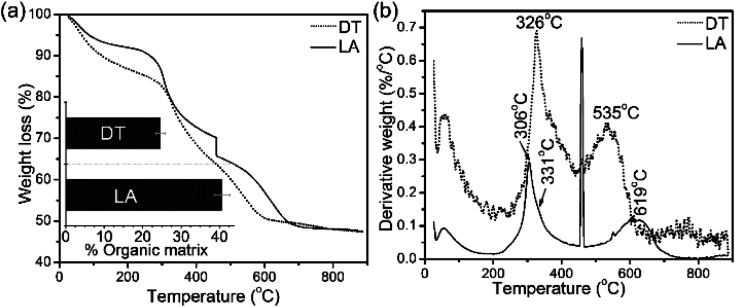
(a) Thermal gravimetric analysis (TGA) data and differential thermal gravimetric (DTG) analysis data (b) of shell materials (LA: *Lingula anatina,* and DT: *Discinisca tenuis*). The bar chart inserted in (a) represents the calculated total shell macromolecule contents in the 200–650 °C range. *L. anatina* and *D. tenuis* contain 40.6 wt% and 24.6 wt% total organic matrix, respectively. See Table 1 and text for further details.

The TGA and DTG data from the shells show weight losses and multistage decompositional steps (Fig. 2 and Table 1). The initial weight loss during TGA, 8.4 wt% for *L. anatina* and 7.6 wt% for *D. tenuis*, occurs between 30° and 200 °C due to the loss of moisture and occluded water molecules.^15^ The second TGA stage of weight loss of 24.6 wt% for *D. tenuis* and 40.6 wt% for *L. anatina* occurs from 200 °C to 650 °C, and is due to the decomposition of organic macromolecules,^38,39^ including collagen and collagen-like materials^40,41^ within the brachiopod shells. The final TGA step shows thermal degradation from 650 °C to 890 °C which is attributed to the loss of carbonate ions (CO_3_^2−^) as CO_2_ from the disintegrated apatitic mineral in the shell biominerals.^38,39^ The weight loss in this region amounts to 3.9 wt% (*L. anatina*) and 1.6 wt% (*D. tenuis*). Concerning CO_3_^2−^, these components are incorporated into the apatitic lattice^42^ as CaCO_3_, but not present as discrete phase in apatitic shell biominerals. The apatite (PO_4_^3−^) to calcium carbonate (CO_3_^2−^) ratio of the samples is shown in Table 1, and is considerably lower for *L. anatina* than *D. tenuis*. The final residue (ash) is interpreted as the apatite content, and is 47.5 ± 2.7 wt% for *L. anatina* and 65.0 ± 3.4 wt% for *D. tenuis*.

**Table 1:** Composition of brachiopod shells as derived from thermal gravimetric analyses^a^

Sample	H_2_O (wt%)	Organic matrix (wt%)	CaCO_3_ content (wt%)	Apatite content (wt%)	Apatite/CaCO_3_ ratio
*L. anatina*	7.6 (1.0)	40.6 (2.3)	3.9 (0.9)	47.5 (2.7)	12.3
*D. tenuis*	8.4 (0.9)	24.6 (1.2)	1.6 (0.6)	65.0 (3.4)	41.1

a Notes: standard deviations are given in parentheses. The occluded water molecules and organic content were determined between 30–200 °C, and 200–650 °C, respectively. The carbonate content was calculated between 650–890 °C. The apatite content is equivalent to the ash content, and was calculated after heating at 900 °C. Note that the carbonate content (CO_3_^2−^) in the carbonated apatitic biominerals is presented as ‘calcium carbonate’ but this is purely formal, and ‘calcium carbonate’ is not present as a discrete phase in these shells.

### 3.2 Structural composition by Raman spectroscopy

**Fig. 3:**
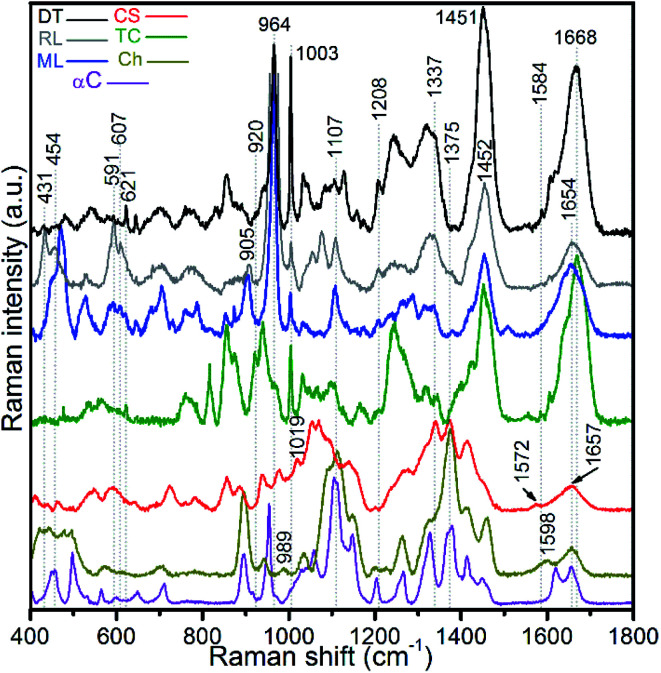
Baseline-corrected Raman spectra of hydrogen peroxide-treated brachiopod shells (modern/living (ML) and recent (RL) *Lingula anatina*, and recent *Discinisca tenuis* (DT)), untreated chondroitin sulfate A (CS; glycosaminoglycan), untreated type I collagen (TC), untreated chitosan (Ch) and untreated α-chitin (αC) acquired using a 633 nm laser. The spectra are normalized. The amide I peak of TC at 1668 cm^−1^ is comparable to the recent *D. tenuis* at 1664 cm^−1^. In contrast, the amide I peak of *L. anatina* was detected at 1654 cm^−1^. The amide I peak of glycosaminoglycan and chitosan was detected at 1657 cm^−1^, but the structure/feature is distinct as compared with the shell matrices. See ESI Table S1† for peak assignments.

Raman spectra of hydrogen peroxide-treated shell materials – modern (ML) and recent (RL) *L. anatina*, and recent *D. tenuis* (DT) – are shown in Fig. 3 and are compared with the spectra of type I collagen, polysaccharides such as chitin/chitosan and chondroitin sulphate A (glycosaminoglycan). The peak positions in the 400–1800 cm^−1^ region are listed in ESI Table S1,† with assignments made by comparison with the literature *e.g.*^21,22,27,28,43–47^ The data reveal spectra attributable to collagen, glycosaminoglycans, lipids and hydroxyapatite components. Some peaks from standard polysaccharides and glycosaminoglycan overlap with the collagenous peaks, but the spectra are quite divergent from one another. The spectra of the shells exhibit an overall strongly similar shape, suggesting a common structural pattern, although subtle variations exist. The most significant of these occurs in the amide I region where the peak of modern and recent *L. anatina* is attributed to the α-helical conformation at 1654 cm^−1^ (Fig. 3), and somewhat similar to the amide I peak of glycosaminoglycan and chitosan at 1657 cm^−1^. In contrast, *D. tenuis* has a peak at 1664 cm^−1^ which suggests the presence of a 3_10_ helix structure, very similar to that of the type I collagen where the peak appears at a slightly higher frequency (1668 cm^−1^). Other differences include the C=C in-plane ring stretch at around 1604 cm^−1^ (tyrosine) and 1584 cm^−1^ (phenylalanine), and the peak at around 1555 cm^−1^ which is assigned to amide II, owing primarily to N–H in-plane bending with a contribution from C–N stretching vibrations. These peaks are evident in *D. tenuis*, but only appear as shoulders in modern and recent *L. anatina* (Fig. 3). An intense and narrow peak at 1003 cm^−1^ is assigned to phenylalanine of collagen, and is very prominent in *D. tenuis* compared with the peaks in *L. anatina* samples (ML and RL). In addition, the 920 cm^−1^ peak in *D. tenuis* is similar to a peak in type I collagen and is associated with the protein side chain vibration of proline. There is no evidence of this peak in either recent *L. anatina* or modern *L. anatina* (Fig. 3 and ESI Fig. S2†), but a rocking vibration of the methyl side chains occurs at 905 cm^−1^. It is possible there is interaction of glycosaminoglycan components with collagen, since amide III peaks in the 1201–1343 cm^−1^ region are prominent in all samples. A CH_2_-wagging of collagen and/or CH_3_ deformation of lipids at around ∼1451 cm^−1^ are prominent in all samples, except in standard glycosaminoglycan. The 1375 cm^−1^ and 1069 cm^−1^ peaks that distinguish polysaccharides including glycosaminoglycan from collagen are extremely weak in the spectra of the shells. A weak ∼1604 cm^−1^ peak and a shoulder at ∼1616 cm^−1^ that correspond to tyrosine (Fig. 3) are not visible in the standard spectra of polysaccharides and glycosaminoglycan.

The *L. anatina* samples (ML and RL) have peaks that are either weak or shift to a slightly higher frequency compared to *D. tenuis*, or *vice versa* (Fig. 3 and ESI Fig. S3†). For example, in *L. anatina*, a carbonate peak at ∼1074 cm^−1^ is observed, with a characteristic shift by 3 cm^−1^ to 1077 cm^−1^ in *D. tenuis*. A shoulder peak at ∼1086 cm^−1^ for *L. anatina* (ESI Fig. S3†) is attributed to an asymmetric stretching mode of P–O phosphate groups, and is assigned at 1084 cm^−1^ in *D. tenuis*. The peaks at 607 cm^−1^, 591 cm^−1^, 580 cm^−1^, 454 cm^−1^ and 431 cm^−1^ (ESI Table S1†) are assigned to the degenerate bending modes of P–O vibrations within the PO_4_^3−^ groups.^21,34^ Triply degenerate asymmetric stretching modes of phosphate at 1053 cm^−1^, 1040 cm^−1^ and 1032 cm^−1^ overlap with the protein skeletal peak *v*C–O component and/or *v*C–O stretching vibrations of the carbohydrate residues in collagen and glycosaminoglycans. A relative intense *v*_1_PO_4_^3−^ mode vibration at 964–965 cm^−1^ was observed for all samples, typical for hydroxyapatite.^34^ The underlying signals centred at 948–950 cm^−1^ (ACP), 955–956 cm^−1^ (OCP) and between 971 and 975 cm^−1^ (TCP) were obtained by fitting the composite Raman peak with a Gaussian function for all samples (ESI Fig. S2†). A peak at 979.5 cm^−1^ was detected in *D. tenuis*, but was not found in the other samples (ESI Fig. S2†). It is possible to associate the signals at ∼980 cm^−1^ with the monohydrogen phosphate P–O bond, as well as with other transient phosphate groups besides ACP and OCP.^24,25,29^

**Fig. 4:**
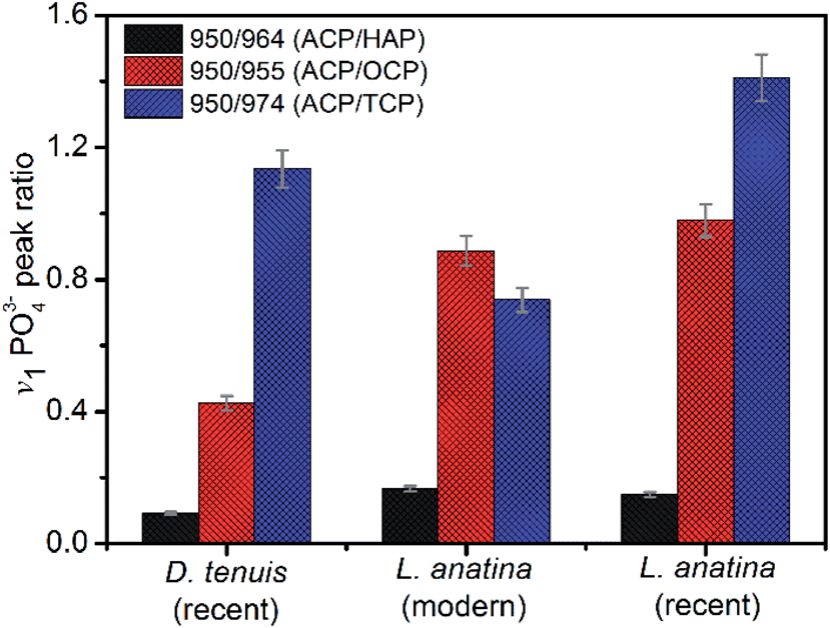
Raman metrics investigated for mineral peak area ratios. Error bars are ±5 standard deviations. Recent *Discinisca tenuis*, recent *Lingula anatina* and modern/living *Lingula anatina*, respectively. ACP = amorphous calcium phosphate, HAP = hydroxyapatite, OCP = octacalcium phosphate and TCP = tricalcium phosphate.

The peak positions and full width measured at half maximum intensity (FWHM) of the intense *v*_1_PO_4_^3−^ stretching vibration permit relative mineral crystallinity of the apatite phase to be determined (ESI Table S2†). Broader peaks reflect lower crystallinity. The fitted 950 cm^−1^ peak for recent *L. anatina* is broad and the FWHM is higher compare to other samples (ESI Fig S2†). The FWHM of other components are comparable to one another. The HAP peak position lies at ∼964 cm^−1^ and the FWHM of the shells is lower, ∼11 cm^−1^. Four *v*_1_PO_4_^3−^ phosphate peaks (Fig. 4) were used to determine three peak area ratios, because the relative abundance of ACP is fairly independent. The ACP : HAP ratios of the shells are low (<0.2). The ACP : OCP ratio of *D. tenuis* is almost half that of the *L. anatina* samples. The ACP : TCP ratio of modern *L. anatina* is lower than that of recent *L. anatina*, possibly due to the lower wavenumber at 971 cm^−1^ compared with the wavenumber of recent *L. anatina* that appears at 975 cm^−1^. Additionally, the FWHM of the 971 cm^−1^ peak is broader than is typically assigned in the other samples (ESI Table S3†). Taken together, the peak positions at about 950 cm^−1^ and 955 cm^−1^ suggest a transition state of ACP and OCP, and could provide quantitative insight into the preservative conditions of apatitic biominerals of modern and/or fossils brachiopod shells.

### 3.3 Structural properties by IR spectroscopy

**Fig. 5:**
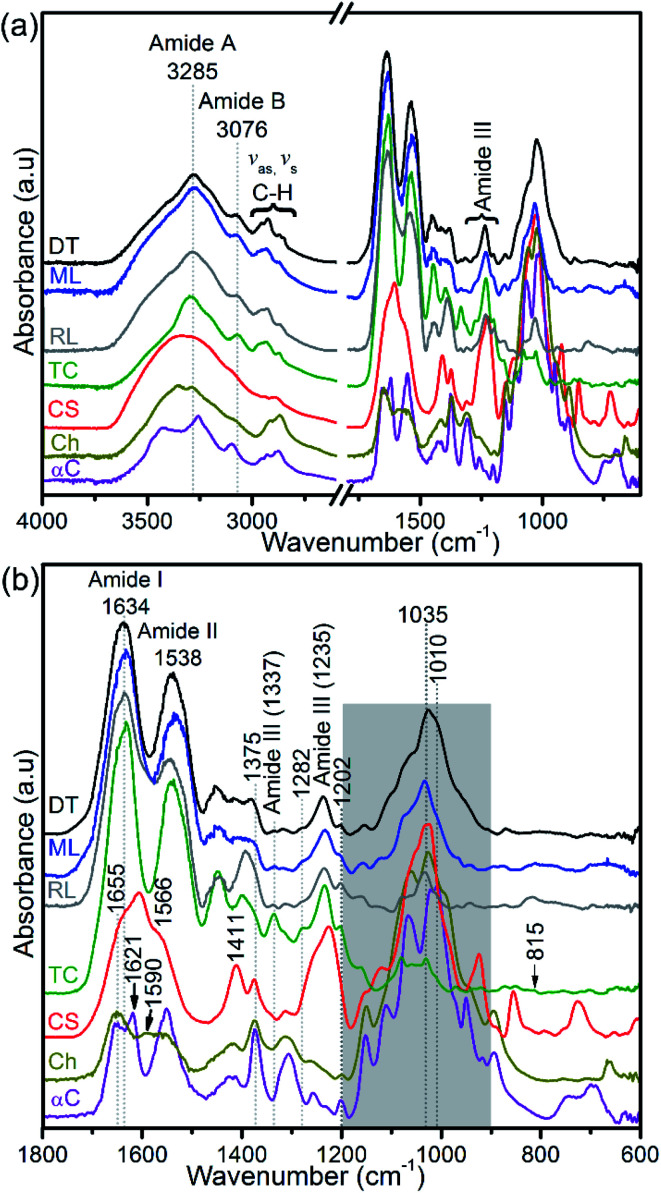
FTIR spectra of hydrogen peroxide-treated brachiopod shells (modern/living (ML) and recent (RL) *Lingula anatina*, and recent *Discinisca tenuis* (DT)), untreated chondroitin sulfate A (CS; glycosaminoglycan), untreated type I collagen (TC), untreated chitosan (Ch) and untreated α-chitin (αC). (a) Shows a larger wavenumber range (4000–600 cm^−1^) than the expanded range (1800–600 cm^−1^) in (b). The collagen amide I and III peaks of the spectra of the shells are related to that of type I collagen. The shaded area in (b) for shell spectra demonstrates PO_4_^3−^ stretching modes of phosphate groups and is depicted in more detail in Fig. 6. Shell spectra are compared with the type I collagen spectrum (Fig. 6) and glycosaminoglycan spectrum (ESI Fig. S4†). For chitin and chitosan spectra, the shaded region is mainly attributed to the C–O stretching and CH_3_ deformation/wagging of polysaccharides. See ESI Table 1† for peak assignments.

Collagen, glycosaminoglycans, lipids, and protein-linked phosphate components in the apatitic shells were identified using FTIR (Fig. 5a). The shell spectra are distinct from that of glycosaminoglycan, chitin and chitosan (Fig. 5b) but similar to that of type I collagen, with the amide A, amide B and C–H peaks at around 3285 cm^−1^, 3076 cm^−1^ and 2973–2850 cm^−1^, respectively. Also, the amides I and II of the standard glycosaminoglycan, chitin and chitosan are distinct compared with the shell and type I collagen spectra. The absorption peak of shells centred at around 1634 cm^−1^ shows a triple-helical conformation (Fig. 5b). The FTIR spectra further support the identification of a prominent population of helical structures of collagen in the 1202–1338 cm^−1^ region (Fig. 5b and ESI Table S1†). The features at 1202 cm^−1^ and 1337 cm^−1^ correspond predominantly to the CH_2_-wagging vibration of the glycine backbone and the proline side chain of the glycine–X–Y sequence structure of collagen.^44,48^ The peaks at 1236 cm^−1^ and in the 1395–1423 cm^−1^ range are assigned to a C–N stretching mode and a symmetrical COO^−^ stretch of collagen and/or the carbonate of biominerals.^45,49^ A 1226 cm^−1^ peak and a shoulder at ∼1255 cm^−1^ that are neither evident in the spectra of shells nor in type I collagen are assigned predominantly to the SO_3_^−^ asymmetric stretching of sulphated glycosaminoglycans,^44,47^ see Fig. 5b.

**Fig. 6:**
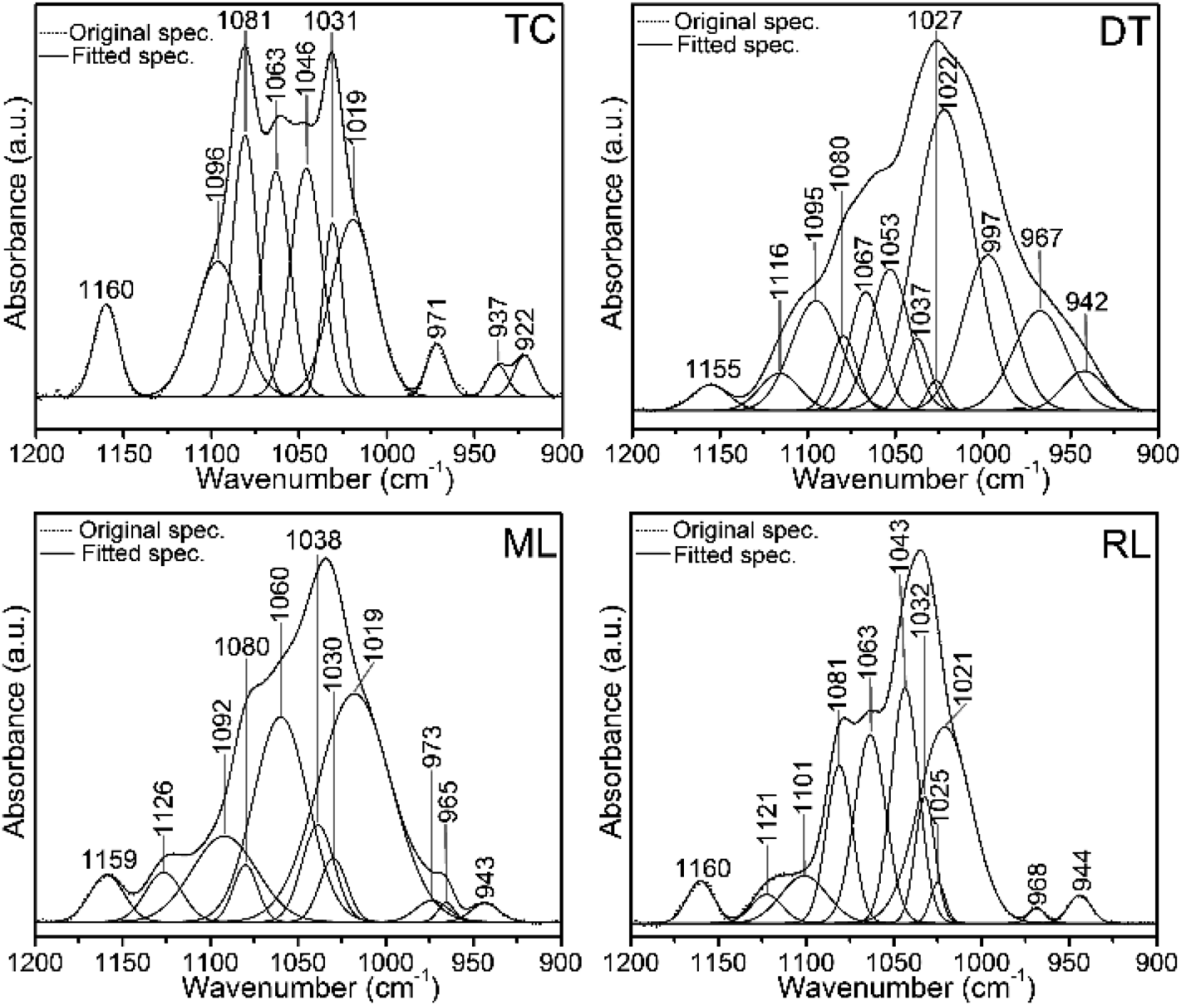
Original FTIR normalized spectra and the corresponding spectral decompositions in the 1200–900 cm^−1^ region of type I collagen (TC) and brachiopod shells (DT, ML and RL). Spectra show protein-linked symmetric and antisymmetric PO_4_^3−^ stretching modes of phosphate groups. Peaks at ∼1020 cm^−1^ and 1030 cm^−1^ denote nonstoichiometric and stoichiometric apatites, respectively. Some of the peaks of the shells overlap with the type I collagen peaks. DT, ML and RL represent recent *Discinisca tenuis*, modern/living *Lingula anatina* and recent *Lingula anatina*, respectively. See Table 2 for peak assignments.

FTIR spectra in the 1200–900 cm^−1^ region contain several useful signals, including the ∼1160 cm^−1^ peak which is attributed to the C–O mode of polysaccharide residues in type I collagen and standard glycosaminoglycan (ESI Fig. S4†), and is present in the phosphatic shells (Fig. 5 and 6). The features of the FTIR spectra of type I collagen in this region are distinct compared with the shells and glycosaminoglycan. Although collagen type I and glycosaminoglycan shared a ∼922 cm^−1^ peak; 1081, 1063, 1046 and 1031 cm^−1^ peaks also occur in this region of the type I collagen spectrum (Fig. 6). In contrast, the shell spectra exhibit a broad peak at 1027 cm^−1^ for *D. tenuis* which is similar to that of glycosaminoglycan at 1027 cm^−1^ (ESI Fig. S4†). This peak shifts to a higher frequency (∼1035 cm^−1^) for both modern and recent *L. anatina*.

Several Ca-phosphate mineral components are suitably fit for the shell matrices (Fig. 6 and Table 2). The peaks at 1126 cm^−1^, 1121 cm^−1^, 973 cm^−1^, 968 cm^−1^ and 965 cm^−1^ for *L. anatina* (RL and ML), and at 1116 cm^−1^, 997 cm^−1^ and 967 cm^−1^ for the recently alive *D. tenuis* arise mainly from the symmetric and antisymmetric PO_4_^3−^ stretching modes of phosphate groups.^26,29,50^ There are other components in the spectra of *L. anatina* at about 1020 cm^−1^, 1025 cm^−1^ and 1030 cm^−1^, and at about 1022 cm^−1^, 1027 cm^−1^ and 1037 cm^−1^ for *D. tenuis* (Fig. 6). It has been hypothesised that the 1025 cm^−1^ peak arises from PO_4_^3−^ attached to collagen fibrils.^51^ The signal at ∼1031 cm^−1^ is indicative of stoichiometric apatites, while the 1020–1022 cm^−1^ peak corresponds to nonstoichiometric apatites containing PO_4_^3−^ and/or CO_3_^2−^.^29,50,52^ The feature at 1101–1092 cm^−1^ for the Ca-phosphate shell samples, which is also assigned in type I collagen and glycosaminoglycan at ∼1096 cm^−1^, is associated with stoichiometric apatites, and is due to the presence of CO_3_^2−^ and/or PO_4_^3−^. The curve-fitting spectra consistently show underlying peaks that are representative of a specific chemical environment and are comparable with one another.

**Table 2:** FTIR spectra (cm^−1^) peak position of stoichiometric and nonstoichiometric phases in the brachiopod shells. The peaks overlap with the (type I) collagen and chondroitin sulfate A (glycosaminoglycan; GAG) peaks^a^

Type I collagen	Chondroitin sulfate A	*D. tenuis*	*L. anatina* (ML/RL)	Assignment
	1122	1116	1126/21	HPO_4_^2−^ stoichiometric apatite overlap with GAGs
1096	1094	1095	1092/1101	CO_3_^2−^ and/or HPO_4_^2−^groups
1081		1080	1080/1	*v*C–O in collagen and GAGs overlaps with *v*_3_PO_4_^3−^
1063	1062	1067	1060/3	*v*C–O in collagen and GAGs overlaps with lipids and *v*_3_PO_4_^3−^
1046		1053	1043	*v*C–O carbohydrate residues in collagen and GAGs/*v*_3_PO_4_^3−^
		1037	1038	PO_4_^3−^groups in OCP
1031	1027	1027	1030/2	*v*C–O carbohydrate residues in collagen and GAGs overlap with *v*_as_PO_4_^3−^ group of stoichiometric apatite
			1025	*v*_as_PO_4_^3−^
1019	1018	1022	1019/21	*v*_as_PO_4_^3−^ and/or CO_3_^2−^ group in nonstoichiometric apatite
	992	997		*v*_as_PO_4_^3−^ in apatite environment overlap with GAGs
971			973	*v*_1_PO_4_^3−^
		967	965/8	*v*_1_PO_4_^3−^

a Note: peaks were derived from second derivative of deconvoluted spectra and their assignments from literature values.^26,29,34,44,50,52^

### 3.4 Analysis of the amide I peak and collagen cross-links

Based on Gaussian functions, FTIR spectra in the 1720–1580 cm^−1^ region were fit to calculate collagen cross-links ratios. This spectral region exhibits several underlying components (ESI Fig. S5†), including a trivalent collagen cross-links peak at 1660–1662 cm^−1^, and a divalent cross-links peak at 1689–1692 cm^−1^.^27^ FWHMs of pyridinoline (trivalent) in the type I collagen are 39 cm^−1^, slightly higher than in the shell biominerals (30 ± 6 cm^−1^). The FWHM of the divalent peak in type I collagen is 21 cm^−1^, which is similar to that of recent *L. anatina* (22 cm^−1^), but is significantly higher than for modern *L. anatina* and *D. tenuis* (12 cm^−1^ and 14 cm^−1^, respectively). The collagen maturity was calculated from the 1660/1690 ratio,^27^ and ranges from 9.9–11.5 for the apatitic shells, comparable to but slightly lower than for the type I collagen which is 13.0 (ESI Table S4†).

## 4. Discussion

The results of this study provide distinct compositional properties for the shells of two representative taxa belonging to separate superfamilies of lingulid organophosphatic brachiopods, *L. anatina* (Linguloidea) and *D. tenuis* (Discinoidea). Most of the differences relate to organic constituents, which supports previous work.^3,8^ Shells of *D. tenuis* are highly mineralized, with 24.6 wt% total organic macromolecules compared with 40.6 wt% for the *L. anatina* shells. The chemical composition of the *D. tenuis* shell is different with respect to mineral content, carbonated apatite, and carbonate content compared to the *L. anatina* shells. The apatite:carbonate ratio of the *D. tenuis* shell is about three fold higher than in shells of *L*. *anatina* (Table 1). However, the external shell surface ultrastructures have rather similar features, in which pores are filled with collagen-like organic fibrils (Fig. 1). Organic fibrils together with Ca-phosphate nanoparticles intercalate and form mineralized compact layers that persist for a very long time (in samples as old as the Cambrian), which eventually lead to fossilized shell biominerals.^3^

MicroRaman spectroscopic measurements reveal that the apatitic minerals and shell-associated macromolecules are essentially similar in component-related information in the FTIR spectra. The spectral peaks of the organic matrix and the apatitic mineral components compare well with type I collagen and previous apatite mineral compositional data.^19–22,28,51,53^ There are some distinct differences in the intensity of peaks between the shells, especially in the Raman spectra. This can be affected by a number of experimental factors, including the different thicknesses of the samples,^54^ the orientation of the biomolecules with respect to the polarised incident beam and the mode of molecular vibration.^43^ The depth resolution of the microRaman technique is expected to be 6 μm, but may vary around the focal plane^33,55^ due to a different adjustment of the laser focus. Another factor is the possibility of other macromolecules accompanied by high mineral crystallinity in the phosphatic shells. Nevertheless, results reveal some prominent *v*_2_PO_4_^3−^ and *v*_4_PO_4_^3−^ phosphate peaks in the spectra of *L. anatina* shells that appear weak in the *D. tenuis* shell. The spectra along with FEG-SEM data reveal that inorganic and organic matrices are entwined in the same layer and appear to be composites. However, in some cases specific habitats are not uniformly mineralised,^6^ and can even be entirely composed of shell macromolecules.^2^

The weight of carbonate of apatitic biominerals (Table 1) correlates with the crystallite size in that it lowers crystallinity^42^ and the areas of different mineral content coexist in the Ca-phosphate shell biominerals. As a general rule, higher biomineral turnover leads to a larger number of sites with a lower degree of mineralization in the biomineral matrix.^56^ In this work, the samples have Raman spectra that are characterized by broad, less well-resolved peaks (ESI Fig. S2†). The results show transient mineral phases other than amorphous Ca-phosphate. Raman analysis, just like XRD, is sensitive to disorder even in crystalline materials.^57^ The Raman spectra enable selective interpretation of amorphous Ca-phosphate (948–950 cm^−1^), octacalcium phosphate (955–956 cm^−1^) and tricalcium phosphate (971–975 cm^−1^). Associated with these is a 1011 cm^−1^ peak that is a P–O stretching vibration of monohydrogen phosphate (HPO_4_^2−^) which is also found in octacalcium phosphate.^24,25^

FEG-SEM measurements reveal nanoparticles that compare well with the amorphous Ca-phosphate reported using transmission- and scanning-electron microscopes.^2^ Watanabe and Pan revealed mixtures of varying amounts of granule-containing apatitic matrices such as dicalcium phosphate dehydrate (brushite) and octacalcium phosphate from columnar cells of the lingulid *Glottidia pyramidata* by using transmission electron microscopy.^58^ It is uncertain if transient octacalcium phosphate, a mineral of relevance in bone mineralisation, has been documented or identified in the shells of *L. anatina* and *D. tenuis*. Tricalcium phosphate is another form of Ca-phosphate that was identified in this study, thus supporting the previous study.^42^

TGA analyses demonstrate a ∼2.5-fold higher wt% of carbonate in the *L. anatina* compared to the *D. tenuis* shells. The wt% carbonate of the samples are comparable with data for vertebrate bones and teeth.^19,34^ Raman spectral results reveal a prominent peak at ∼1105 cm^−1^ associated with type-A carbonate substitution (CO_3_^2−^ for OH^−^) in the hydroxyapatite lattice. The peaks at 1074–1077 cm^−1^ and components at 671–679 cm^−1^ and 714–730 cm^−1^ are attributed to type-B carbonate, where CO_3_^2−^ ions occupy the PO_4_^3−^ sites.^21,34,53^ In the FTIR spectra, there are numerous carbonate and/or phosphate peaks, even in the case of simple stoichiometric apatite and nonstoichiometric apatite, consistent with the inorganic components in mammalian skeletal tissues (Table 2). Notable, for instance, is the peak at ∼1030 cm^−1^, which occurs in stoichiometric apatite, whereas a nonstoichiometric apatite peak at ∼1020 cm^−1^ probably indicates the persistence of vacancies on the crystals, and seems consistent with the composition of other Ca-phosphate mineral phases, *e.g.*^34,50^ Raman spectra show the degenerate stretch of HPO_4_^2−^ ions at 1125–1134 cm^−1^ which can also be detected in the FTIR spectra at 1116–1126 cm^−1^.^50^ While these authors interpret these peaks to be consistent with several non-apatitic phosphates such as octacalcium phosphate, the peaks in this region overlap with some polysaccharide such as chitin/chitosan and/or glycosaminoglycan components (ESI Table S1†). Strong ionic interactions are expected between glycosaminoglycans and proteins to modulate biomineral processes.^59,60^ Some collagenous peaks overlap with the glycosaminoglycans due to a variety of different types of interactions, including hydrogen bonds and hydrophobic interactions with the sugar backbone.^60^ These interactions are not unusual for the organic macromolecules of phosphatic hybrid composite biominerals.^3,21^

Generally, the collagen amide I peak in the 1720–1580 cm^−1^ region is a polymer composite of several partially resolved components,^27^ see ESI Table S2.† Based on the analyses of the structural protein of collagen, the most crucial components at about 1660 cm^−1^ and 1690 cm^−1^ are shown to be proportional to the relative amounts of mature (trivalent) cross-linked pyridinoline and the divalent (immature) cross-linked dihydroxylysinonorleucine.^21,27^ These moieties have been extensively identified by FTIR and Raman spectroscopic methods, and hence are used intensively to determine the maturity state of the cross-linking network in the bone collagen fibril.^19,20,27^ The computational method, based on the Gaussian function, determined the presence of the above mentioned moieties in both Raman and FTIR spectral data, thus suggesting the incorporation of cross-linked collagen in organophosphatic brachiopod shells. For the first time this study demonstrates the presence and relative abundance of collagen and its cross-linking ratio in recent organophosphatic shells. The values in the range of 9.9–11.5 agree closely with one another (with marginal variation), and are only somewhat lower in abundance relative to the amount of 13.0 in type I collagen (ESI Table S4†).

This study proposes that covalent cross-linking of an organic collagen network is an important feature in the biostructural and mechanical properties of organophosphatic brachiopod shells. Previous work has proposed a role for intermolecular collagen cross-linking during the development of underlying hybrid composite matrices,^20^ and has suggested it is essential for transient Ca-phosphate precursor formation and crystal growth during ontogeny.^20,27,61^ While this claim has yet to be conclusively demonstrated for brachiopod shells, it is one of many adaptations, both molecular and ultrastructural, that influence the overall mechanical properties of bioceramic–biopolymer composites in biomineralic aggregates.^62^

Here, for the first time, the results of non-destructive analyses show that as well as glycosaminoglycans and chitin, type I collagen is an important component in the organic-biomineral matrix of the shells of two species of organophosphatic linguloid brachiopods. The presence of type I collagen is even more prevalent in the *D. tenuis* shells, due to the amide peaks that are closely comparable to type I collagen. This supports previous reports of hydroxyproline and proline in brachiopod shells that also correlate with a collagen matrix.^6,14^ A large amount of alanine and a low amount of glycine^3,14^ supports an amorphous type of collagen in *Lingula* shells.^6^ The data reported here do not enable quantification of the proportion of amino acids in the shell matrix, but the spectra reveal prominent hydroxyproline and proline peaks that are known to stabilize the helical structure of collagen, which strengthens apatite hybrid composite materials.^63^ In this study, a distinctive Raman peak at 905 cm^−1^ for the *L. anatina* shells (RL and ML; Fig. 3) does not appear to be directly comparable with the peaks in the *D. tenuis* shell and type I collagen. Such a peak was also observed in the Raman spectrum of poly(alanine)^64,65^ and extracellular fibrous silk protein with unique characteristics of strength and elasticity.^17^ In cases where this peak is observed, it is predominantly assigned to a combination of C^α^–C and C–N stretching modes of the backbone nuclei and a rocking vibration of the alanyl.^17,64,65^

The relative intensities of the 905 cm^−1^ peak in the Raman spectra of *L. anatina* shells vary (Fig. 3), but in general the peak is less prominent compared to the one reported in fibrous silk protein.^17^ These authors attributed the high intensity of the ∼905 cm^−1^ peak to the longer alanine sequences, and a secondary structure that consisted of a β-sheet conformation at ∼1668 cm^−1^. In contrast, the amide I position of *L. anatina* is very dissimilar in this same region, with the main peak assigned to α-helix at 1654 cm^−1^ (Fig. 3). Based on the secondary structure of the repeating units of poly(alanine), a shorter region could easily adopt an α-helical conformation.^66^

*Lingula anatina* may have independently undergone domain combinations to produce extracellular matrix biomineralization and possess lineage-specific (poly)alanine-rich fibres,^16^ as compared with *Discinisca tenuis*.^3,14^ Species in the genus *Lingula* are infaunal, living in a burrow, whereas *Discinisca* is a shallow marine epibenthic form.^67^ Epifaunal *Discinisca* shells attach to hard substrates by a muscular pedicle, whereas the burrowing of *Lingula* is accomplished by complex motions of the valves.^67^ The differences in the total amount of organics and the apatite/calcium carbonate ratio as revealed by the thermal gravimetric analyses reported in this study support the variation in the chemical compositions of shell biominerals between the two species, suggesting natural selection of the most appropriate inorganic–organic biocomposites to fulfil their ecological habitus.

Taken together, the mineralized biopolymers of shell biominerals are typically made of a protein–polysaccharide matrix. Admixtures of protein biominerals with various polysaccharides achieve various conformations according to their chemistry and chemical environment.^59,68^ Individual polysaccharides, such as glycosaminoglycans, differ from each other by the type of hexosamine, and the position and configuration of the glycosidic linkages.^59^ The repeat sequence patterns of the protein motif of collagen could be glycine–proline–X or glycine–X–hydroxyproline, where X may be any other amino acid. Although a glycine residue in the repeated pattern of the extracellular matrix is invariant, a previous study replaced obligate glycine with d-alanine in globular proteins, and discovered that d-amino acids can significantly increase stability of the protein motif.^69^ It may be that shells of *L. anatina* employed hydrophobic (poly)alanine along with a crystalline matrix (in its thinner laminated layers of fibrous organic), in order to increase flexibility and reduce brittleness.^2^ While a collagen matrix had been proposed in the shells of *Lingula* in previous studies,^5,6,14^ the current study compliments these findings by synchronously determining more precisely the components of brachiopod shells including organic and inorganic matrices, and shows for the first time the typical extracellular matrix in the shells of *D. tenuis*. As previously proposed, the main organic constituents of the shells are glycosaminoglycans, chitin and non-collagenous proteins, albeit there is less certainty about the form and distribution of these components within studied brachiopod shells.^5–7^

## 5. Conclusions

This study provides critical new understanding of the chemical and structural components of organophosphatic brachiopod shells, which are some of the earliest biomineralizing bilaterian animals that appeared in the Cambrian explosion and are still living.

The chemical composition of the shells of *L. anatina* is distinct compared to the *D. tenuis* shell. For instance, the total amount of organic macromolecules in *L. anatina* shells is 40.6 wt% compared with 24.6 wt% for the *D. tenuis* shell. In contrast, the weight percentage of carbonate content of the shell biominerals are comparable with data for vertebrate skeletons.

FEG-SEM has shown organic fibrils that intercalate with the Ca-phosphate mineral. Synchronous spectroscopic analyses provide for the first time strong organic–inorganic signals and evidence for collagen, and the interactions with the glycosaminoglycan components, as compositional constituents of brachiopod shells.

Based on the Gaussian function fits, careful analyses of the microRaman and ATR-FTIR spectra show that the organophosphatic brachiopod shells consist of transient amorphous Ca-phosphate and octacalcium phosphate as well as tricalcium phosphate.

Non-destructive spectroscopic methods – microRaman and ATR-FTIR spectroscopies, and destructive TGA analyses – are excellent techniques to determine or monitor the conservation of the fossilised and/or modern shell macromolecules and mineral components in organophosphatic brachiopod shells. These techniques require almost no special sample preparation, in contrast to many other methods that require significant manipulation of sample preparations, including chemical fixation and epoxy resin that influences and/or contaminate organic biopolymers.

## Conflicts of interest

There are no conflicts to declare.

## Supplementary Material

RA-010-D0RA07523J-s001
