# Supplementary material for: Characterization of organophosphatic brachiopod shells: spectroscopic assessment of collagen matrix and biomineral components
Source: RSC Adv. 2020 Oct 20;10(63):38456–67. doi: 10.1039/d0ra07523j (PMC9057340; doi:10.1039/d0ra07523j)
Supplement: RA-010-D0RA07523J-s001 [file RA-010-D0RA07523J-s001.pdf]

Electronic Supplementary Material (ESI) for RSC Advances.
This journal is © The Royal Society of Chemistry 2020

Electronic Supplementary Information

Characterization of organophosphatic brachiopod shells: spectroscopic assessment of collagen matrix and biomineral components†

Oluwatoosin B. A. Agbaje,*[a-c] Simon C. George,[b] Zhifei Zhang,[d] Glenn A. Brock,[c,d] Lars E. Holmer[a,d]

[a] Department of Earth Sciences, Palaeobiology, Uppsala University, Uppsala, Uppsala, Sweden

[b] Department of Earth and Environmental Sciences and MQ Marine Research Centre, Macquarie University, Sydney, Australia.

[c] Department of Biological Sciences, Macquarie University, Sydney, Australia.

[d] State Key Laboratory of Continental Dynamics, Shaanxi Key Laboratory of Early Life & Environments, Department of Geology, Northwest University, Xi'an, 710069, China.

*** Correspondence:** toosin.agbaje@mq.edu.au; toosin91014@gmail.com

SI Table 1a. Wavenumber from Raman and FTIR spectra of type I collagen and hydrogen peroxide-treated brachiopod shells.

Type 1 Collagen		Chondroitin sulfate A		*D. tenuis*		*L. anatina* (ML/RL)		Assignment/Component
Raman	FTIR	Raman	FTIR	Raman	FTIR	Raman	FTIR	
1668				1664				Amide I, β-sheet/3_{10} helix, Collagen
1657						1654		Amide I, α-helix, GAGs/Collagen
	1634		1635*		1637		1634/6	Amide I, Triple helix, Collagen/GAGs
*1616				1616		*1616		Amide I, Collagen
1604			1606	1606		1604		Amide I
1584				1585		1585*		νC=C ring of phenylalanine
		1572						GAGs
1555	1544		1566*	1557	1538	1543*	1538/44	Amide II
1451	1450	1448*		1451	1455	1452	1454/47	wCH$_2$/δasCH$_3$/Phospholipids
1423<br>1398	 1401	 1414	 1411		1417	1422	 1403/1395	νCOO$^-$
		1375	1375	1384*	1380	1379w		ν_sCH$_3$ of GAGs
1336/43	1337	1341		1337	1338	1336	1337/8	Amide III, α-helix, Collagen/GAGs
1317	1317	1316*	1310	1319	1318	1313	1317/5	Amide III, α-helix
	1281	1277		1289w	1282	1286	1282	Amide III, α-helix
1265		1267*		1265		1264		Amide III, α-helix, Collagen/GAGs
1245	1235	1237*		1242	1237	1238/42	1236	Amide III, β-sheet and/or random coils,
			1226/55					Sulfate asymmetric stretching of GAGS
1208		1201w		1205		1206/7		νC-C of tryptophan and phenylalaline
	1202				1202		1203	wCH$_2$ from Collagen
1165/74				1172		1174		νC-O-C, Collagen
	1161	1151*	1155	1157	1156	1155	1158/60	νC-O-C of polysaccharide (GAGs)
1125		1138	1123	1126		1134	1125/6	νC-C of collagen/C-O-S of GAGs overlaps with the HPO$_4^{2-}$
1097		1090		1104	1108*	1107	1116	ν_1CO$_3^{2-}$; A-type
1086*	1080			1084		1086*	1080	νC-O in collagen and GAGs overlaps with ν_3PO$_4^{3-}$
1078*				1077*		1072/4		ν_1CO$_3^{2-}$; B-type
1067	1061	1069	1062*	1064*	1065*		1069*/4	νC-C-O of carbohydrate in collagen and GAGs overlaps with lipids
		1055				1053		GAGs overlap with ν_3PO$_4^{3-}$
1046				1040		1040		νC-O of carbohydrate in collagen and GAGs/ν_3PO$_4^{3-}$
1031	1031		1027	1032	1025	1032	1035/6	νC-O of protein; ν_3PO$_4^{3-}$ overlaps with proline νC-C
		1019		1012*		1011*		GAGs overlap with HPO$_4^{2-}$
1003				1004		1002/3		νC-C of phenylalanine (Collagen)
965	971	978	985*	964	952	964/5	968/71	ν_{as}PO$_4^{3-}$(hydroxyapatite); νC-O-S
				942	944*	942	943/4	νC-C backbone of collagen
939	937	939	937*	939		936		νC-O-C backbone of collagen/GAGs
920	922		924	921				νC-O-C backbone of collagen/GAGs
						905		νC$^{\alpha}$-C, νC-N, rCH$_3$
		885	891*	887			895/7	δC-H for anomers (GAGs)
874	875			877	870	872/3	875/3	νC-C of hydroxyproline
853	852	855	855	855	856*	852/3		νC-C of proline (Collagen)/GAGs
				827/33				νC-COO$^-$ of tyrosine (Collagen)
815	815			818	810*	814	814/9	νC-O-C collagen crosslink
785		783	793*	778		786/76		ν(C-C)/pyrimidine ring breathing
759		760		758		758/60		δC-COO$^-$/ν_4CO$_3^{2-}$; B-type
		722	726	714,<br>701		730,<br>704		ν_1CO$_3^{2-}$; B-type<br>ν_1CO$_3^{2-}$; B-type
		693		671		679/7		v$_4$CO$_3^{2-}$
		642	655	642		643		tC-C, wCOO$^-$ of Collagen/GAGs

621				621		619		*t*C-C Collagen
				602		607		$v_4PO_4^{3-}$
590*		590		591<br>579		591, 580		$v_4PO_4^{3-}$ overlaps with GAGs
563		547				556		*v*(S-S) in collagen/GAGs
534				536		527		*v*(S-S) in collagen
476		461/92		476		470		*v*(S-S) in collagen/GAGs
				450		450/4		$v_2PO_4^{3-}$
		439		431		431		$v_2PO_4^{3-}$ overlaps with GAGs
		410						GAGs

Notes: modern/living (ML) and recent (RL) *Lingula anatina* shells. Chondroitin sulfate A represents sulphated glycosaminoglycan

SI Table 1b. Raman and FTIR band assignment for pure α-chitin and chitin (organic) fibre

α-chitin		Chitosan		Assignment
Raman	FTIR	Raman	FTIR	
1655	1656	1657	1651	Amide I, α-helix
1617	1621	1598	1590	Amide I, *v*C=O
	1553		1562	Amide II
1447		1460		CH_2, CH_3
	1428		1422	CH_2
1413	1415	1410		*vs*COO^-
1376	1376	1374	1376	*r*C-CH_2, *δ*C-CH_3
1327		1323	1315	Amide III, *v*CH_3
	1308			Amide III, CH_2 w,
1265	1260	1263	1260	Amide III, *v*C-H, *δ*N-H, *δ*C=O
	1234	1225	1235	Amide III, N-H ib, C-N s
1202	1204	1202	1202	Amide III, C-O-H d
1147	1154	1146	1151	*v*C-O-C
1111/04	1113	1111		*v*C-O-C, ring
	1068	1087		*v*C-O-H
1057			1061	*v*C-O, *v*C-C, ring
1031	1024	1036	1027	*δ*C-O-H, *v*C-O, *v*C-C skeletal
	1010	989	996	*δ*C-O-H, *v*C-O
971*				*δ*CH_3, *v*C-O
953	952	942	951	*δ*CH_3, *δ*C-O-H
915	919			CH_3
895	895	895	895	*δ*CH_x, C-O-C glycosidic bond
				C-C, C-O, C-H
				C-C, C-O, C-H
	746			CH_2
710	701	707		C-O, C-H, N-H
	692			C-O, C-H, N-H
648	634		660/7	C-C, C-O, C-H
598				C-C, C-O, C-H,
				PO_4^{3-} s
563		570		C-C, O-H
530				C-C skeletal backbone
496		495/78		C-C skeletal backbone
450/5		441		C-C-C ring
		420		C-C-C ring

SI Table 2. Assignment of amide I peak positions and percentage areas to secondary structure.

Sample			
	FTIR	Area %	Assignment
Type 1 Collagen (TC)			β-turn
	1692	4	β-turn
	1661	51	β-sheet, 3_{10} helix
	1651	12	α-helix
	1644	4	Unordered
	1636	6	3_{10}-helix
	1629	23	3_{10}-helix
	1616		Side chain, Tyrosine and tryptophan
Recent *D. tenuis* (DT)			β-turn
	1692	3	β-turn
	1678	13	β-sheet
			β-sheet
	1660	34	β-sheet, 3_{10}-helix
	1650/8	2	α-helix
	1643	22	Unordered
	1631	26	3_{10}-helix
			β-sheet
			β-sheet; Side chain
	1616		Side chain, Tyrosine and tryptophan
	1603		Side chain, tyrosine
	1595		Side chain, Phenylalanine
Modern *L. anatina* (ML)			
			β-turn
	1692	2	β-turn
	1680	7	
	1662	23	β-sheet, 3_{10}-helix
	1652	29	α-helix
			Unordered
	1635	32	3_{10}-helix
	1626	7	β-sheet
	1616		Side chain, Tyrosine and tryptophan
	1603		Side chain, tyrosinate

	1591		Side chain, Phenylalanine
Recent *L. anatina* (RL)			β-turn
	1689	6	β-turn
			β-turn
	1660	54	β-sheet, 3_{10}-helix
	1652	6	α-helix
	1640/6	5	Unordered
	1631	29	3_{10}-helix
			β-sheet
	1615		Side chain, Tyrosine and tryptophan
	1600		Side chain, tyrosinate
	1591		Side chain, Phenylalanine

Notes: ML = modern, RL = recent.

SI Table 3. A. Raman spectra (cm^{-1}) peak position of the phosphate group in brachiopod shells.

D. tenuis		*L. anatina* (ML)		*L. anatina* (RL)		Component
Shift	FWHM	Shift	FWHM	Shift	FWHM	
948	7	949	8	950	14	ACP [1]
955	8	955	7	955	8	OCP [1, 2]
958	2			959	3	CAP [2, 3]
964	11	964	10	965	11	HAP [2, 3]
973	7	971	12	975	10	TCP [1, 3]
~980	5					DCPD [3]

Notes: ML = modern, RL = recent, FWHM = full width measured at half maximum intensity, ACP = amorphous calcium phosphate, OCP = octacalcium phosphate, CAP = carbonated hydroxyapatite, HAP = hydroxyapatite, TCP = tricalcium phosphate, DCPD = Dicalcium phosphate dihydrate.

SI Table 4. Collagen crosslinks 1660/1690 ratio of type I collagen and brachiopod shells.

	Type I collagen	*D. tenuis*	*L. anatina* (ML)	*L. anatina* (RL)
Collagen cross-links	13.0±0.53	11.5±0.47	11.0±0.42	9.9±0.39

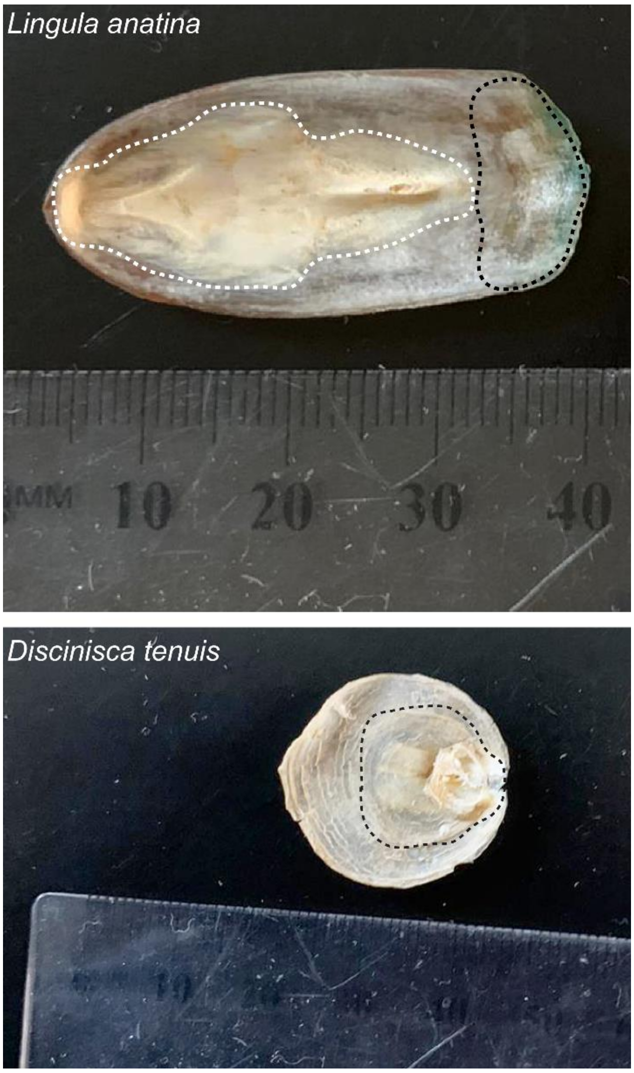

SI Figure 1. *Lingula anatina* and *Discinisca tenuis* showing the interior part of the shells. The white dashed line in *Lingula anatina* and the black dashed region in *Discinisca tenuis* indicate part of the shell that was exempted from vibrational spectroscopy analyses and SEM imaging. Black dashed line in *Lingula anatina* denotes the region that was measured for microRaman and Fourier Transform Infrared.

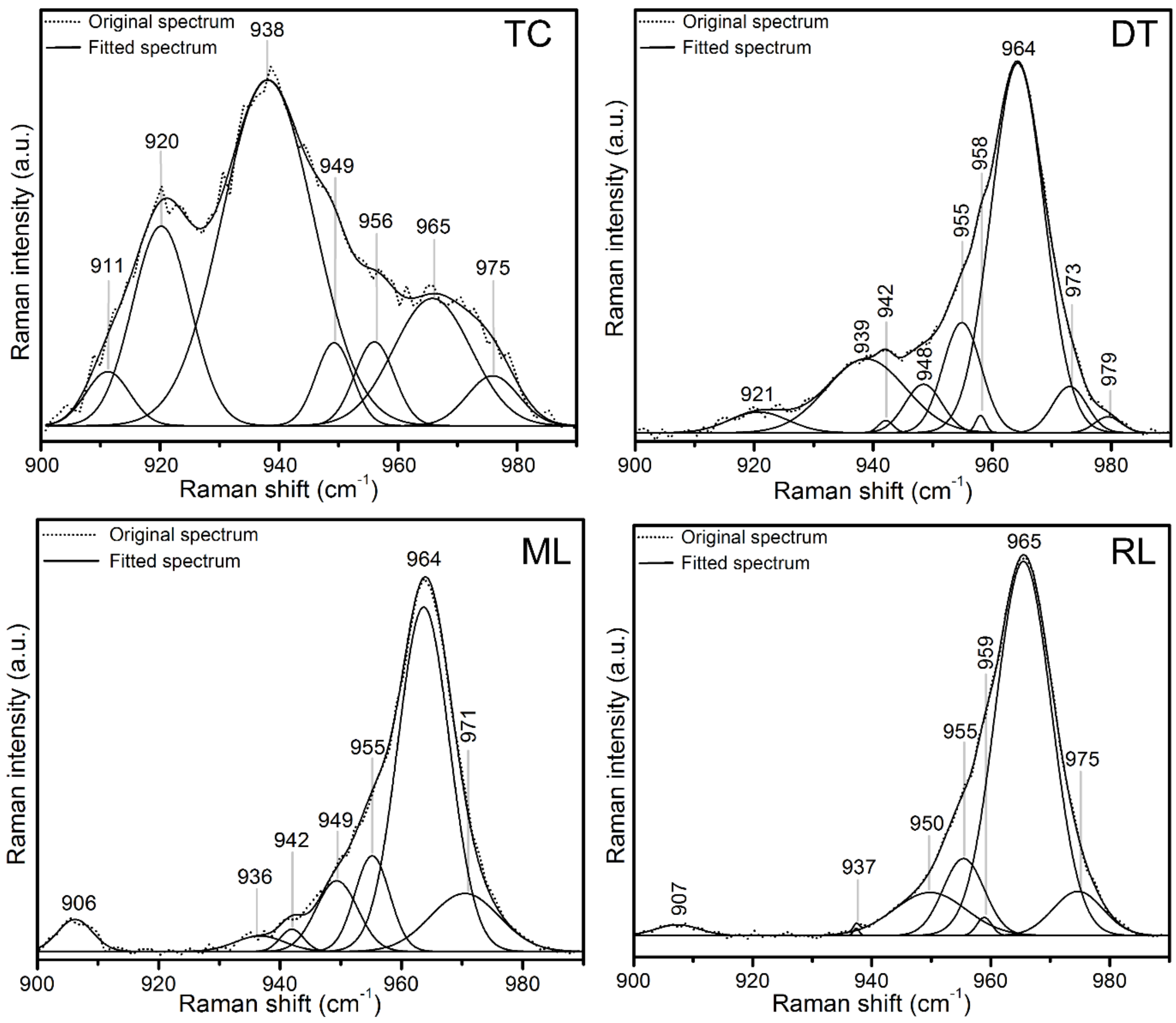

SI Figure 2: Raman spectra of type I collagen (TC) and shells of brachiopods (DT, ML and RL) in the 900–990 cm^{-1} region. The Raman data were deconvoluted by a Gaussian fit. See text for the interpretation of the peaks. DT, ML and RL represent *Discinisca tenuis*, recent *Lingula anatina* and modern/living *Lingula anatina*, respectively.

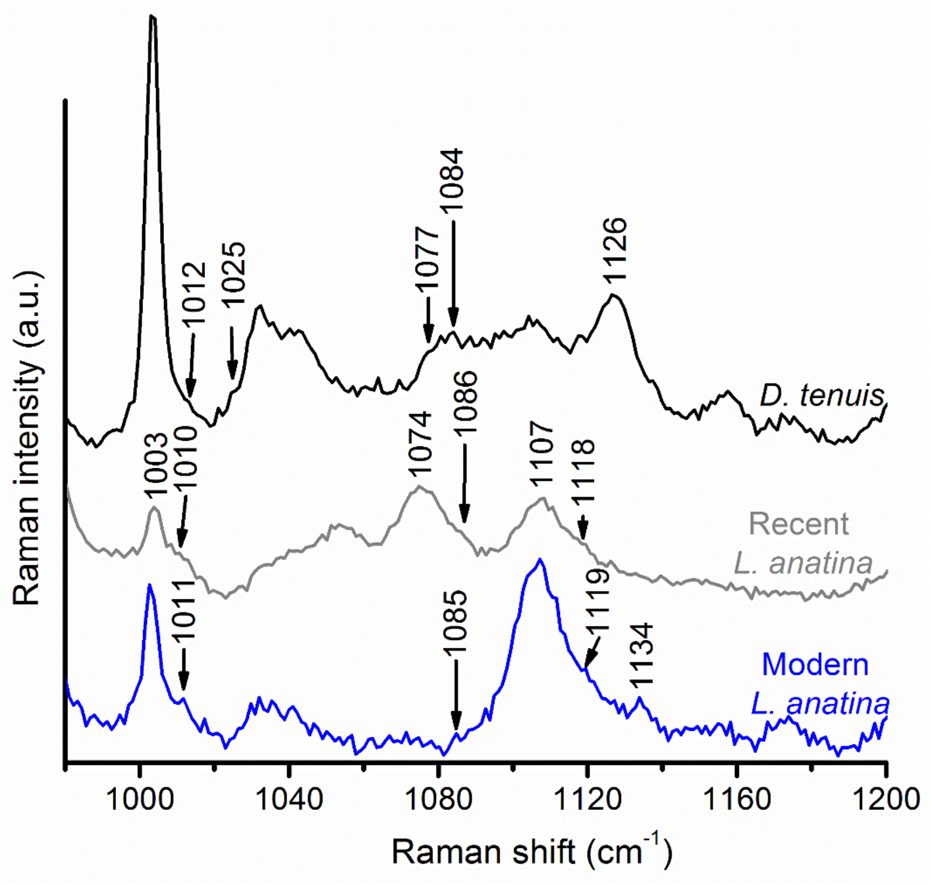

SI Figure 3: Raman spectra of brachiopods shells in the 980–1200 cm^{-1} region.

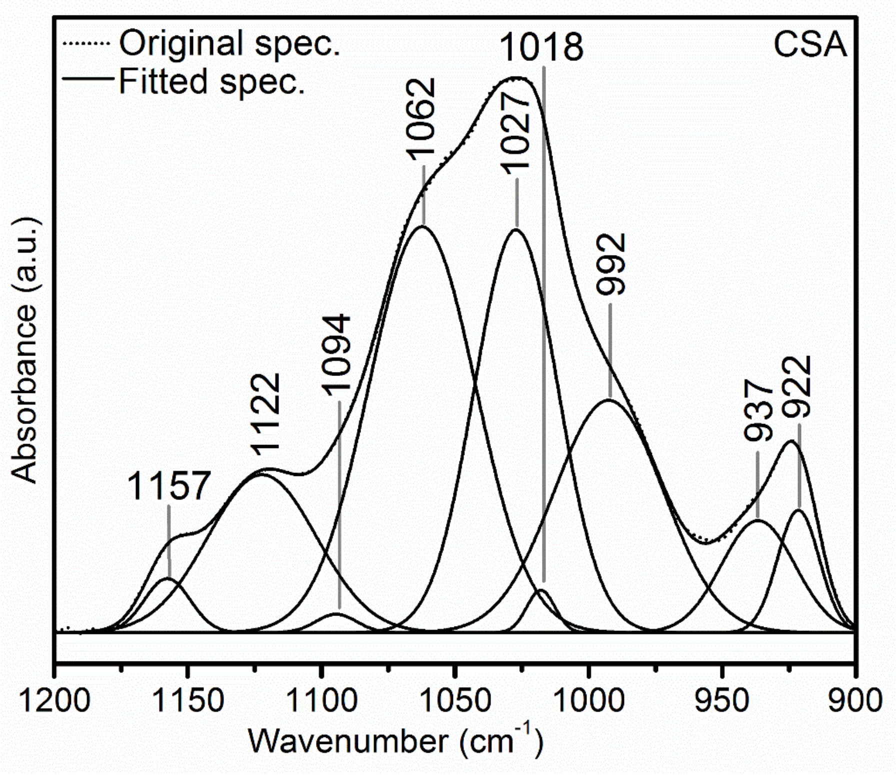

SI Figure 4. Curve-fitting of FTIR spectrum of Chondroitin sulphate A (CSA; glycosaminoglycan) in the 1200–900 cm^{-1} region. See **Table 2** for peak assignments.

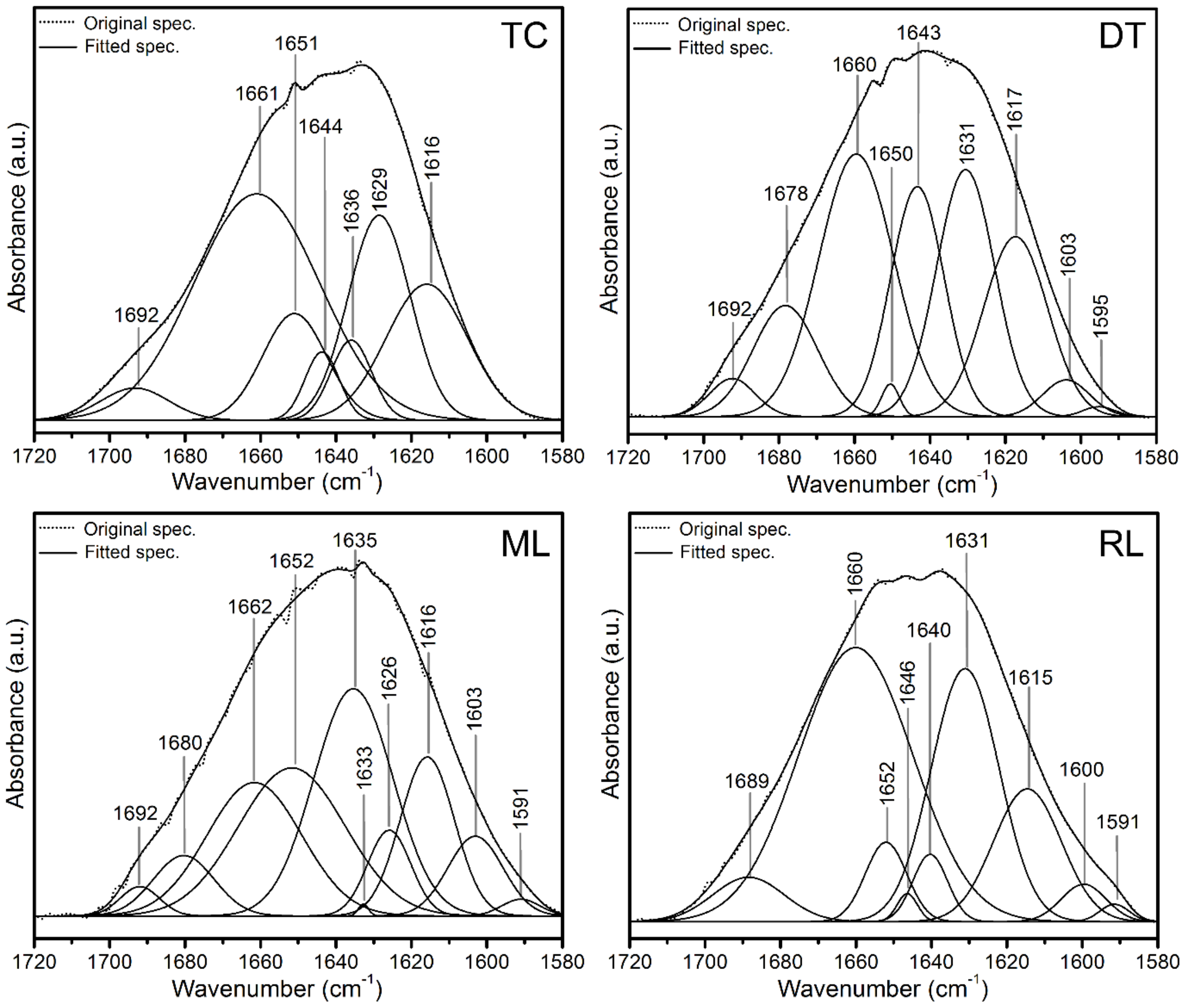

SI Figure 5: Curve-fitting of FTIR spectra of amide I in the 1720–1580 cm^{-1} region. TC, DT, ML and RL represent type I collagen, *Discinisca tenuis*, recent *Lingula anatina* and modern/living *Lingula anatina*, respectively.

References

1 N. J. Crane, V. Popescu, M. D. Morris, P. Steenhuis, M. A. Ignelzi Jr, *Bone*, 2006, **39**(3) 434-442.

2 J. Freeman, B. Wopenka, M. Silva, J. Pasteris, *Calcif. Tissue Int*. 2001, 1, 68(3).

3 S. Koutsopoulos, *J. Biomed. Mater. Res. A,* 2002, **62** 600-612.
